# Mycotoxin binder improves growth rate in piglets associated with reduction of toll-like receptor-4 and increase of tight junction protein gene expression in gut mucosa

**DOI:** 10.1186/s40104-017-0210-4

**Published:** 2017-11-01

**Authors:** Linghong Jin, Wei Wang, Jeroen Degroote, Noémie Van Noten, Honglin Yan, Maryam Majdeddin, Mario Van Poucke, Luc Peelman, Anne Goderis, Kurt Van De Mierop, Ronny Mombaerts, Stefaan De Smet, Joris Michiels

**Affiliations:** 1 Department of Applied Biosciences, Ghent University, Valentin Vaerwyckweg 1, 9000 Ghent, Belgium; 2 Laboratory for Animal Nutrition and Animal Product Quality, Department of Animal Production, Ghent University, Coupure Links 653, 9000 Ghent, Belgium; 3 Department of Nutrition, Genetics and Ethology, Faculty of Veterinary Medicine, Ghent University, Heidestraat 19, 9820 Merelbeke, Belgium; 4 Nutrex, Achterstenhoek 5, 2275 Lille, Belgium

**Keywords:** Binder, Deoxynivalenol, Gut barrier, Gut health, Mycotoxin, Pigs

## Abstract

**Background:**

Deoxynivalenol (DON) is a mycotoxin produced by *Fusarium* species in the field, commonly found in cereal grains, which negatively affects performances and health of animals. Mycotoxin binders are supposed to reduce the toxicity of mycotoxins.

**Method:**

The effect of a mycotoxin binder (containing acid-activated bentonite, clinoptilolite, yeast cell walls and organic acids) on growth performance and gut health was studied. Hundred and twenty weaning piglets were allocated to 4 treatments, with 5 pens of 6 piglets each, arranged in a 2 × 2 factorial design: control diet; control diet with 1 kg/t binder; control diet with DON; and control diet with DON and 1 kg/t binder. From d0–14, the diet of DON-challenged groups was artificially contaminated with a mixture of DON (2.6 mg/kg), 3-acetyl-deoxynivalenol (0.1 mg/kg) and 15-acetyl-deoxynivalenol (0.3 mg/kg), after which the total contamination level was reduced to 1 mg/kg, until d37. On d14, one pig from each pen was euthanized and distal small intestinal mucosa samples were collected for the assessment of intestinal permeability, and gene expression of tight junction proteins, toll-like receptor 4, inflammatory cytokines and intestinal alkaline phosphatase.

**Results:**

After 37 d, there were no differences in growth performance between control and DON-challenged groups (*P* > 0.05). Nevertheless, groups that received diets with binder had a significantly higher average daily gain (ADG) and average daily feed intake (ADFI) for the first 14 d as well as for the whole period, compared to groups without binder (*P* ≤ 0.05). Groups with binder in the diet also exhibited lower expression of toll-like receptor 4 in distal small intestinal mucosa at d14, compared to groups without binder (*P* ≤ 0.05). Interestingly, comparing the two DON treatments, piglets fed DON and binder had significantly higher ADFI and ADG compared to those with only DON for the first 14-d (*P* ≤ 0.05). Addition of binder to DON contaminated diets, also down-regulated the gene expression of toll-like receptor 4 (*P* ≤ 0.05) and increased mRNA level zona occludens 1 (*P* ≤ 0.10) as compared to DON.

**Conclusions:**

The present data provide evidence that the binder improves growth rate in piglets associated with reduction of toll-like receptor-4 and increase of tight junction protein gene expression. However, the current study does not allow to assess whether the effects of the binder are mediated by alterations in the toxicokinetics of the mycotoxin.

## Background

The contamination of feedstuffs with mycotoxins is a worldwide issue. Mycotoxins are harmful secondary metabolites of fungi which can cause intoxications at very low dosage. Deoxynivalenol (DON) is a type B trichothecene mycotoxin, produced by *Fusarium* species. DON is noted for two typical toxicological effects: reduced feed intake and induction of emesis in swine. Under natural conditions, DON is present along with its two major acetylated forms, 3-acetyl-deoxynivalenol (3A–DON) and 15-acetyl-deoxynivalenol (15A–DON) at lower concentrations than DON in cereals [1]. DON is physically stable and can easily enter the food chain [2]. Humans and all animal species can exhibit toxic effects after exposure to DON [3], with pigs being the most susceptible species [4].

The intestinal mucosa is constantly challenged by various chemical and biological contaminants, and functions as a vital barrier between the intestinal milieu and the luminal content [5, 6]. After consumption of feed contaminated with DON, the intestine can be exposed to high levels of the toxin [7]. Studies show that DON can affect the intestinal histology and morphology by affecting intestinal cell viability and proliferation. DON is also able to regulate the production of pro-inflammatory cytokines, increasing the expression of interleukin 1 beta (IL-1β), interleukin 2 (IL-2) and interleukin 6 (IL-6) in the jejunum, and IL-1β, IL-6 and tumor necrosis factor alpha (TNF-α) in the ileum in pigs [8]. An in vitro study indicated that DON is able to decrease transepithelial electrical resistance (TEER) and increase the permeability of IPEC-1 cells in a dose and time dependent manner [7]. The alterations in these two parameters are related to the decreased expression of specific tight junction proteins (TJPs) 9 [7, 9]. In the intestinal epithelium, the activation of mitogen-activated protein kinase (MAPK) by DON and its acetylated derivatives suppresses the expression of TJPs, which is responsible for the loss of barrier function [10, 11].

In order to solve the problem caused by mycotoxicosis, various strategies have been developed, including physical, chemical, and biological methods [12, 13]. The most common approach is the addition of mycotoxin binders to feeds [14]. Mycotoxin binders are large weight molecules, capable of binding to mycotoxins in animal feeds. These binder-mycotoxin complexes pass through the gastrointestinal tract without dissociating, preventing mycotoxin uptake [15]. The complex passes through the GIT and is excreted via the faeces, thereby helping to minimize absorption of mycotoxins by target organs and alleviating the adverse effects of mycotoxins.

DON was the most prevalent single mycotoxin found in all feedstuffs all over the world in 2015 [16]. However, there are few publications about the effects of toxin binders on gut health in piglets. This study was conducted to assess the effect of addition of a mycotoxin binder to the feed on gut health and performance in pigs following a 37-d dietary exposure to DON.

## Methods

### Animals and dietary treatments

An animal feeding experiment in a 2 × 2 factorial design with either or not addition of DON, and either or not addition of binder to the feed was performed. A total of 120 weaning (24 d of suckling period) piglets with an average weight of 7.3 kg were used in this study. They were provided with water and feed ad libitum throughout the experiment. Animal experimental procedures were in accordance to the guidelines of the Ethical Committee of the Faculty of Veterinary Sciences, Ghent University, Belgium.

Piglets were randomly allocated to 4 dietary treatments. Each treatment contained 5 pens with 6 piglets per pen. The 4 treatments were as follows: CON, negative control diet (uncontaminated basal diet); CON + BIN, negative control diet with 1 kg/t mycotoxin binder; DON, negative control diet with DON; DON + BIN, negative control diet with DON and 1 kg/t mycotoxin binder. The mycotoxin binder was a blend of indigestible adsorbents that bind mycotoxins in the GIT (Free-Tox, Nutrex NV, Belgium). It contains acid-activated bentonite, clinoptilolite, yeast cell walls and organic acids. From d 0 until d 14 (sampling on d 14) of the experiment, the diet of DON and DON + BIN was artificially contaminated with a mixture of DON (2.6 mg/kg), 3A–DON (0.1 mg/kg) and 15A–DON (0.3 mg/kg), after which the DON contamination level was reduced to 1 mg/kg from d 14 until d 37. The composition of the negative control wheat-barley-soybean based diets is given in Table 1. The DON-challenge diets were artificially contaminated with a fungal culture containing DON and its metabolites. DON was produced in vitro by *F. graminearum*. After growing up of the mold, the amount of DON was quantified by ELISA assay on the medium. The result was verified by LC/MS/MS on the certified standard blank wheat [17]. Results showed that the medium contained 240 mg/kg total DON metabolites (87.5% DON, 2.7% 3A–DON and 9.8% 15A–DON). All other mycotoxins were under the detection limit. Based on the concentration in the medium, the amount of medium needed for 3 mg/kg and 1 mg/kg in pre-starter and starter diets, respectively, was calculated. After homogenization, the medium was mixed into the basal diet. Three mg/kg for pre-starter period (from d 0 until d 14) was chosen because typically feed contamination with 2–5 mg/kg DON is required to induce reduction of feed intake and decrease of body weight gain [18]. It was further reduced to 1 mg/kg for the starter period (from d 14 until d 37) as model for chronic exposure.

**Table 1:** Composition of the negative control diet (CON) for pre-starter (d0-d14) and starter (d14-d37) periods

Pre-starter		Starter	
Ingredients	%	Ingredients	%
Wheat	22.57	Barley	25.00
Barley	22.50	Wheat	22.93
Whey	7.00	Corn	15.00
Extruded soybeans	2.40	Toasted soybeans	12.00
Calcium formiate and lactic acid	1.00	Whey	4.20
Potato protein	2.00	Extruded soybeans	1.70
Toasted soybeans	12.00	Calcium formiate and lactic acid	0.50
Extruded oats and barley	10.00	Coconut	0.20
Corn	7.50	Soybean meal, CP49	9.07
Soybean meal, CP49	4.04	Wheat gluten feed	2.69
Fat, > 88% triglycerides	0.50	Beet pulp, sugar 72%	2.00
Sodium bicarbonate	0.30	Fat, > 88% triglycerides	0.95
Organic acids mixture^a^	0.30	Organic acids mixture^a^	0.30
Lime fine	0.29	Salt	0.05
Premix^b^	7.60	Premix^c^	3.40
Composition		Composition	
NE, kcal/kg	2350	NE, kcal/kg	2350
Crude protein, g/kg	173	Crude protein, g/kg	178
Crude fibre, g/kg	37	Crude fibre, g/kg	38
Calcium, g/kg	5.2	Calcium, g/kg	7.9
Phosphorus, g/kg	4.5	Phosphorus, g/kg	4.8
Sugar + Starch, g/kg	457	Sugar + Starch, g/kg	438

^a^Organic acids mixture: contains formic acid, phosphoric acid and citric acid

^b^Providing per kg of complete diet: Vitamin A, 15,000 IU/kg; Vitamin D_3_ 2000 IU/kg; Vitamin E, 200 IU/kg; Vitamin K_3_, 4.0 mg; Vitamin B_1_, 3.0 mg; Vitamin B_2_, 8.0 mg; Vitamin B_3_, 20 mg; Vitamin B_6_, 6.0 mg; Vitamin B_12_, 50.0 μg; niacinamide, 40.0 mg; folic acid, 2.0 mg; biotin, 0.3 mg; Cu, 155 mg/kg; Fe, 150 mg/kg; Mn, 49 mg/kg; Zn, 104 mg/kg; I, 1.55 mg/kg; Se, 0.40 mg/kg

^c^Providing per kg of complete diet: Vitamin A, 15,000 IU/kg; Vitamin D_3_ 2000 IU/kg; Vitamin E, 102 IU/kg; Vitamin K_3_, 4.0 mg; Vitamin B_1_, 3.0 mg; Vitamin B_2_, 8.0 mg; Vitamin B_3_, 20 mg; Vitamin B_6_, 6.0 mg; Vitamin B_12_, 50.0 μg; niacinamide, 40.0 mg; folic acid, 2.0 mg; biotin, 0.3 mg; Cu, 155 mg/kg; Fe, 150 mg/kg; Mn, 49 mg/kg; Zn, 80 mg/kg; I, 1.49 mg/kg; Se, 0.40 mg/kg

### Sampling

After 14 d of feeding, one pig out of each pen was euthanized by intra-peritoneal pentobarbiturate overdose. The GIT was removed and the small intestine was exposed for sample collection. A 10-cm segment from the 75% length of the small intestine (distal small intestine) was collected for Ussing chamber measurements following flushing with saline to remove residual content. Another 20 cm segment from the same region was flushed with saline, placed on a cold plate and slit longitudinally. Then, mucosa was harvested by scraping with a glass-slide followed by snap freezing and storage at −80 °C pending gene expression analysis.

### Growth performance

The weight of piglets as well as the feed intake per pen were determined at d0, d14 and d37. Average Daily Gain (ADG, g/d), Average Daily Feed Intake (ADFI, g/d) and Feed to Gain ratio (F:G) were calculated for periods d0-d14, d14-d37 and d0-d37. Diarrhoea and mortality were daily checked and recorded.

### Ex vivo measurement of intestinal permeability

Permeability was assessed ex vivo in Ussing chambers by measuring the permeability for the macromolecular marker fluorescein isothiocyanate-dextran 4 (FD4, molecular weight 4 kDa) across sheets of mucosa as described by Wang et al. [19]. Briefly, fresh segments of mucosa samples from the distal small intestine (75% of the total small intestinal length) were separated from the seromuscular layer and mounted in the Ussing chamber system. Intestinal sheets were bathed in 6.5 mL Ringer buffer solution with 6 mmol/L glucose and 6 mmol/L mannitol in the serosal and mucosal sides, respectively. The system was maintained at 37 °C and oxygenated (95% O_2_ and 5% CO_2_). After a 20-min equilibration period, 0.8 mg/mL FD4 (Sigma-Aldrich, Bornem, Belgium) was added to the mucosal side. Samples from the serosal compartment were taken at 20 min intervals for 80 min to monitor mucosal-to-serosal fluxes of FD4. Fluorescence intensity of FD4 was determined by fluorescence spectrophotometry (Thermo Fisher Scientific, Marietta, OH, USA). The flux over the 100 min period was calculated and expressed as an apparent permeability coefficient as described before [19].

### RNA isolation and reverse-transcription quantitative real-time PCR

Relative mRNA expression of TJPs (*ZO-1*, *ZO-2*, *OCLN*, *CLDN-1*, *CLDN-2*, *CLDN-5*, *CLDN-7*) and pro-inflammatory cytokines (*TNF-α*, * IFN-γ*, * IL-1β*, *IL-8*), toll-like receptor 4 (*TLR-4*) and a brush border enzyme intestinal alkaline phosphatase (*IAP*) were determined by reverse transcription quantitative real-time PCR (RT-qPCR) and performed according to the MIQE guidelines. Briefly, mucosal total RNA was extracted using the Bio-Rad Aurum Total RNA Fatty and Fibrous Tissue Kit (Bio-Rad Laboratories, Inc., Hercules, CA, USA) according to the manufacturer’s instructions, including an on-column DNase I treatment to remove genomic DNA (gDNA). The concentration and purity (OD_260/280_) of RNA were measured with the NanoDrop ND-1000 (NanoDrop Technologies, Thermo Scientific, Wilmington, DE, USA). 1 μg RNA was analyzed by 1% agarose gel electrophoresis to check RNA integrity (28S and 18S rRNA bands). In addition to this assessment, a minus-RT control PCR was performed using *YWHAZ* as primer to verify the absence of any gDNA contamination. Following this,1 μg of high quality DNA-free RNA was reverse transcribed in the 20 μL reverse-transcription reaction with the ImProm-II cDNA synthesis kit (Promega, Madison, WI, USA), containing both oligo dT and random primers. The obtained cDNA was diluted 10 times with molecular grade water and a control PCR using 2 μL cDNA was performed to verify the reverse-transcription reaction.

Primers (Table 2) used for genes in the study were designed with Primer3Plus. The repeats, the secondary structure and single nucleotide polymorphism in target sequence were checked with RepeatMarker, mfold and dbSNP, respectively. All these primer sequences were gene isoform specific as they were designed based on certain exon-exon boundaries of published pig gene sequences corresponding to the accession number. Primers were then purchased from IDT (Integrated DNA Technologies, Leuven, Belgium).

**Table 2:** Primer sequences used for reverse-transcription quantitative real-time PCR

Gene symbol^a^	Accession number	Nucleotide sequence of primers, 5′-3’		Product length, bp	Tm, °C
		Forward	Reverse		
*CLDN-1*	NM_001244539.1	TATGACCCCATGACCCCAGT	GCAGCAAAGTAGGGCACCTC	108	59
*CLDN-2*	NM_001161638.1	TTCCTCCCTGTTCTCCCTGA	CACTCTTGGCTTTGGGTGGT	152	62
*CLDN-5*	NM_001161636.1	GTGGTCCGCGAGTTCTACGA	CTTGACAGGGAAGCCGAGGT	171	60
*CLDN-7*	NM_001160076.1	GGTCCCCACAAACGTGAAGTA	TCACTCCCAGGACAAGAGCA	114	60
*HPRT-1*	DQ178126	CCGAGGATTTGGAAAAGGT	CTATTTCTGTTCAGTGCTTTGATGT	181	60
*IAP*	XM_003133729.3	GGCCAACTACCAGACCATCG	CCGACTTCCCTGCTTTCTTG	116	60
*IFN*-*γ*	NM_213948.1	GCTTTTCAGCTTTGCGTGACT	CACTCTCCTCTTTCCAATTCTTCA	166	58
*IL-1β*	NM_214055.1	GCACCCAAAACCTGGACCT	CTGGGAGGAGGGATTCTTCA	143	58
*IL-8*	XM_003361958.3	TGTCAATGGAAAAGAGGTCTGC	CTGCTGTTGTTGTTGCTTCTCA	100	60
*OCLD*	NM_001163647.2	CATGGCTGCCTTCTGCTTCATTGC	ACCATCACACCCAGGATAGCACTCA	129	65
*PPIA*	NM_214353	CTGAAGCATACGGGTCCTGG	TGCCCTCTTTCACTTTGCCA	139	65
*TBP*	DQ178129	GATGGACGTTCGGTTTAGG	AGCAGCACAGTACGAGCAA	124	59
*TLR-4*	NM_001113039.2	TTCTTGCAGTGGGTCAAGGA	GACGGCCTCGCTTATCTGAC	135	58
*TNF-α*	NM_214022.1	CATGATCCGAGACGTGGAGC	AACCTCGAAGTGCAGTAGGC	151	62
*ZO-1*	XM_003480423.3	ATCTCGGAAAAGTGCCAGGA	CCCCTCAGAAACCCATACCA	172	61
*ZO-2*	XM_005660148.2	CCAGGAAGCACAGAATGCAA	AAGTCTGGCGGGACCTCTCT	148	61

^a^ *CLDN-1* claudin-1, *CLDN-2* claudin-2, *CLDN-5* claudin-5, *CLDN-7* claudin-7, *HPRT-1* hypoxanthine phosphoribosyltransferase 1, *IAP* intestinal alkaline phosphatase, *IFN-Ƴ* interferon gamma, *IL-1β* interleukin 1 beta, *IL-8* interleukin 8, *OCLN* occludin, *PPIA* peptidylprolyl isomerase A, *TBP* TATA-binding protein, *TLR-4* toll-like receptor 4, *TNF-α* tumor necrosis factor alpha, *ZO-1* zona occludens 1, *ZO-2* zona occludens 2

The RT-qPCR was performed on the CFX96 Touch Real-Time PCR Detection System (Bio-Rad Laboratories, Inc.). Briefly, 2 μL cDNA template, 5 μL 2× KAPA SYBR FAST qPCR Kit Master Mix (Kapa Biosystems, Inc., Wilmington, MA, USA), 2 μL molecular grade water, 0.5 μL forward primer and 0.5 μL reverse primer (5 μmol/L each) were added to a total volume of 10 μL. The amplification conditions were as follows:1) enzyme activation and initial denaturation (95 °C for 3 min); 2) denaturation (95 °C for 20 s) and annealing/extension and data acquisition (annealing temperature depending on primer for 40 s) repeated 40 cycles; and 3) dissociation (melt curve analysis from 70 to 90 °C with 0.5 °C increment every 5 s).

Primers used in this study were first optimized by gradient quantitative real-time PCR. A 5-fold dilution series (5 points, from 1 times to 625 times dilution) of cDNA as standard curve was included at 3 gradient temperatures to determine PCR amplification efficiency and specificity. The standard curve was also included in each run to determine PCR efficiency. In this study, PCR amplification efficiencies were consistently between 90% and 110%. Gene-specific amplification was verified by agarose gel electrophoresis and melting curve analysis. Efficiency was used to convert the Cq value into raw data with the highest expressed samples (lowest Cq value) as a calibrator for the normalization of raw data. The relative expression was expressed as a ratio of the target gene to the geometric mean of three stable expressed reference genes (*PPIA*, *HPRT1* and *TBP*) [19].

### Statistical analysis

After determination of normality and variance homogeneity, a general linear model with the fixed effects of mycotoxin and binder, and the interaction term was used with Tukey’s test as a multiple comparison test in SAS Enterprise Guide 7 (SAS Institute, Cary, NC, USA). *P* ≤ 0.05 was considered as significant. All data are expressed as mean ± standard errors.

Principal component analysis (PCA) as described by Montagne et al. was conducted to work out the variables that contributed most to the variation between subjects [20, 21]. In brief, the data of 17 variables were standardised before the application of PCA. At first, a scree plot was carried out to fix the number of principal components to be maintained. Five principal components were retained with the eigenvalues >1.0. In addition, variables that had a correlation coefficient between variable and all principal components ≤0.5 were excluded. Then, retained variables were grouped into families to check the correlation. Only the main representative variable with highest principal component loading, together with high correlation (*r* > 0.55; *P* ≤ 0.05) within family was retained for the final analysis. Finally, 11 variables entered the final PCA.

## Results

### Growth performances

Only few pigs from different groups had diarrhoea problems in the first week of the experiment, likely following weaning stress. No case of emesis or mortality were observed. Overall, no clinical signs of toxicity were found. There were no significant differences between control groups (CON and CON + BIN) and DON-challenged groups (DON and DON + BIN) regarding growth performances (Table 3). In contrast, pigs supplemented with binder (CON + BIN and DON + BIN) consumed more feed (265 g/d vs. 242 g/d) and had a higher growth (197 g/d vs. 170 g/d) for the first 14-d when compared to pigs that received diets with no binder (CON and DON) (*P* ≤ 0.05). Similarly, for the whole experimental period d0-d37, groups receiving diets with binder (CON + BIN and DON + BIN) showed an improved ADG (368 g/d vs. 341 g/d) and ADFI (548 g/d vs. 519 g/d) compared to groups that received diets without binder (CON and DON) (*P* ≤ 0.05). Meanwhile, There was a trend that groups that received diets with binder (CON + BIN and DON + BIN) had a lower F:G compared to groups that received diets without binder (CON and DON) from d 1 until d 14 of the experiment (*P* ≤ 0.10). Interestingly, within DON-challenged piglets, addition of the binder improved performance in the first 14-d of the experiment; DON contaminated diet supplemented with binder (DON + BIN) showed higher ADFI compared to diet only contaminated with DON (DON) (272 g/d vs. 227 g/d) (*P* ≤ 0.05). This again resulted in a higher growth rate for treatment DON + BIN than treatment DON for period d0–14 (205 g/d vs. 159 g/d) (*P* ≤ 0.05). Also, pigs supplemented with binder (DON + BIN) showed higher body weight at d14 compared to pigs that received diets with no binder (DON) (10.18 kg vs. 9.63 kg) (*P* ≤ 0.05).

**Table 3:** Growth performance (body weight, BW; average daily gain ADG; average daily feed intake, ADFI, and feed:gain ratio, F:G) for periods d0-d14 (pre-starter), d14-d37 (starter) and d0-d37 (total) of piglets fed diets with or without mycotoxins, and with or without binder (*n* = 5)

Item	Treatment^1^								*P*-value		
	CON		CON + BIN		DON		DON + BIN		Mycotoxin	Binder	Mycotoxin × Binder
	Mean	SE	Mean	SE	Mean	SE	Mean	SE			
Day 0–14 (pre-starter)											
BW d0, g	7.30	0.01	7.31	0.01	7.32	0.01	7.30	0.01	0.52	0.85	0.49
BW d14, g	9.82^ab^	0.13	9.96^ab^	0.15	9.63^b^	0.13	10.18^a^	0.12	0.97	0.02	0.05
ADG, g/d	181^ab^	10	188^ab^	11	159^b^	10	205^a^	9	0.80	0.02	0.03
ADFI, g/d	256^ab^	10	257^ab^	12	227^b^	10	272^a^	10	0.50	0.05	0.05
F:G	1.43	0.04	1.38	0.05	1.43	0.04	1.33	0.04	0.64	0.08	0.25
Day 14–37 (starter)											
BW d37, g	20.3	0.43	20.4	0.49	19.9	0.43	21.0	0.40	0.95	0.21	0.35
ADG, g/d	446	17	469	17	444	15	466	13	0.86	0.17	0.55
ADFI, g/d	724	21	760	23	716	21	750	19	0.68	0.11	0.44
F:G	1.59	0.03	1.62	0.03	1.62	0.03	1.62	0.03	0.68	0.55	0.83
Day 0–37 (total)											
ADG, g/d	344	14	366	14	337	12	370	11	0.90	0.05	0.21
ADFI, g/d	527	14	548	16	511	14	549	13	0.59	0.05	0.19
F:G	1.50	0.03	1.50	0.03	1.51	0.03	1.49	0.02	0.84	0.63	0.92

Means with different superscripts (a, b) within row represent differences among treatments (*P* ≤ 0.05)

^1^ *CON* negative control diet (uncontaminated basal diet), *CON + BIN* negative control diet with 1 kg/t mycotoxin binder, *DON* negative control diet with DON, *DON + BIN* negative control diet with DON and 1 kg/ton mycotoxin binder

### Permeability measurements in distal small intestine

**Fig. 1:**
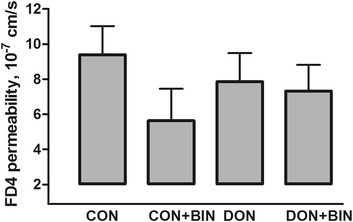
Intestinal permeability for FD4 in distal small intestinal mucosa of piglets fed diets at d14 post-weaning. Data are means ± SE (*n* = 5). *P* for factor mycotoxin is 0.96, for factor binder 0.21, and for mycotoxin × binder 0.51. CON, negative control diet (uncontaminated basal diet); CON + BIN, negative control diet with 1 kg/t mycotoxin binder; DON, negative control diet with DON; DON + BIN, negative control diet with DON and 1 kg/t mycotoxin binder

Neither mycotoxin level, nor binder addition affected FD4 fluxes across distal small intestinal sheets (*P* > 0.05) (Fig. 1). The mean of control groups (CON and CON + BIN) was 7.5 × 10^−7^ cm/s; while the average value of DON-challenged groups (DON and DON + BIN) was 7.6 × 10^−7^ cm/s. On binder level, the difference of FD4 flux between groups that received diets with the addition of binder (CON + BIN and DON + BIN) and groups that received diets without the addition of binder (CON and DON) was larger compared to the difference between DON-challenged and DON-control groups but still not significant.

### mRNA expression of tight junction proteins, inflammatory cytokines and brush border enzyme in distal small intestine

The gene expressions of TJPs (*ZO-1*, *ZO-2*, *OCLN*, *CLDN-1*, *CLDN-2*, *CLDN-5*, *CLDN-7*), pro-inflammatory cytokines (*TNF-α*, *IFN-γ*, *IL-1β*, *IL-8*), *TLR-4* and *IAP* in distal small intestine are described in Table 4. Ingestion of diets contaminated with or without DON, did not change the gene expression of TJPs, pro-inflammatory cytokines, and IAP in distal small intestine (*P* > 0.05), whereas adding the binder to the diets down-regulated the expression of TLR-4 (0.72 for CON + BIN and DON + BIN vs 1.00 for CON and DON; *P* ≤ 0.05). At the same time, there was a trend that groups that received the diet with the addition of binder (CON + BIN and DON + BIN) up-regulated the expression of CLDN-1 compared to groups that received diet without the addition of binder (CON and DON) (*P* ≤ 0.10). More specifically, *TLR-4* gene expression was down-regulated in the DON contaminated diet supplemented with mycotoxin binder (DON + BIN) compared to DON (0.57 vs 1.11; *P* ≤ 0.05). At the same time, there was a tendency that DON + BIN up-regulated the expression of *ZO-1* compared to DON (*P* ≤ 0.10).

**Table 4:** Relative mRNA expression of tight junction proteins, pro-inflammatory cytokines and intestinal alkaline phosphatase in distal small intestinal mucosa of piglets fed diets with or without mycotoxin contamination, and with or without binder at d14 post-weaning (n = 5)

Item^1^	Treatment^2^								*P*-value		
	CON		CON + BIN		DON		DON + BIN		Mycotoxin	Binder	Mycotoxin × Binder
	Mean	SE	Mean	SE	Mean	SE	Mean	SE			
*ZO-1*	1.50	0.16	1.45	0.18	1.29	0.16	1.88	0.15	0.50	0.12	0.08
*ZO-2*	1.40	0.17	1.18	0.17	1.13	0.17	1.42	0.15	0.95	0.82	0.50
*OCLN*	1.09	0.24	0.97	0.24	0.83	0.24	1.60	0.22	0.45	0.18	0.13
*CLDN-1*	1.10	0.17	1.30	0.15	1.03	0.15	1.36	0.15	0.96	0.10	0.36
*CLDN-2*	1.68	0.28	1.17	0.28	0.87	0.28	1.25	0.25	0.19	0.81	0.26
*CLDN-5*	0.95	0.23	1.04	0.23	0.76	0.23	0.66	0.21	0.22	0.98	0.61
*CLDN-7*	1.83	0.23	1.36	0.23	1.20	0.23	1.62	0.21	0.42	0.92	0.26
*TLR-4*	0.88^ab^	0.12	0.87^ab^	0.12	1.11^a^	0.14	0.57^b^	0.11	0.77	0.04	0.05
*TNF-α*	0.98	0.20	0.84	0.20	0.85	0.20	0.98	0.18	0.99	0.98	0.92
*IFN-γ*	0.38	0.18	0.30	0.18	0.73	0.18	0.45	0.17	0.18	0.34	0.41
*IL-1β*	0.97	0.24	0.92	0.24	1.20	0.24	0.79	0.22	0.83	0.34	0.65
*IL-8*	1.48	0.31	1.00	0.31	0.89	0.31	1.20	0.28	0.54	0.78	0.57
*IAP*	0.91	0.22	1.00	0.22	0.73	0.22	1.03	0.20	0.72	0.37	0.65

Means with different superscripts (a, b) within row represent differences among treatments (*P* ≤ 0.05)

^1^ *CLDN-1* Claudin 1, *CLDN-2* Claudin 2, *CLDN-5* Claudin 5, *CLDN-7* Claudin 7, *IAP* Intestinal alkaline phosphatase, *IFN-γ* Interferon, gamma, *IL-1β* Interleukin 1 beta, *IL-8* Interleukin 8, *OCLN* Occludin, *TLR-4* Toll like receptor 4, *TNF-α* Tumor necrosis factor alpha, *ZO-1* Zona occludens 1, *ZO-2* Zona occludens 2

^2^ *CON* negative control diet (uncontaminated basal diet), *CON + BIN* negative control diet with 1 kg/t mycotoxin binder, *DON* negative control diet with DON, *DON + BIN* negative control diet with DON and 1 kg/t mycotoxin binder

### Principal component analysis

BWd0, BWd14, ADG d0-d14, FD4 permeability, ZO-1, ZO-2, OCLN, CLDN-1, CLDN-2, CLDN-5, CLDN-7, TLR4, TNF-α, IFN-γ, IL-1β, IL-8 and IAP were the 17 variables used in the PCA. After application of a first PCA, 5 principal components were retained following a scree plot. BWd0 was the only variable that did not show high correlation on any principal component and was excluded. Then, BWd14 and ADG d0-d14 were grouped into growth performance family, ZO-1, ZO-2, OCLN, CLDN-1, CLDN-2, CLDN-5 and CLDN-7 were grouped into TJPs family, and TNF-α, IFN-γ, IL-1β and IL-8 were grouped into inflammatory cytokines family. FD permeability, TLR-4 and IAP were considered single representatives and were retained for final analysis. Some variables were highly correlated within family. In growth performance family, ADG d0-d14 was highly correlated with BWd14 (*r* = 0.966, *P* ≤ 0.01), yet ADG d0–14 was not retained. Within the family of TJPS, OCLN was correlated with ZO-1 (*r* = 0.762, *P* ≤ 0.01), ZO-2 (*r* = 0.811, *P* ≤ 0.01) and CLDN-7 (*r* = 0.683, *P* ≤ 0.01). Then, OCLD, CLDN-1, CLDN-2 and CLDN-5 were retained for the final PCA. For the family of inflammatory cytokines, only IFN-γ was excluded. Finally, 11 variables were kept for this final PCA (Table 5). The 5 principal components explained 85.5% of the variance, of which the first principal component contributing 22.6% and the second principal component contributing 22.2%. The first principal component grouped the TJPs family members OCLN, CLDN-1, CLDN-2 as well as TNF-α and brush border enzyme IAP together. Principal component 1 had higher principal component score in groups with addition of binder (CON + BIN and DON + BIN) compared to groups without addition of binder (CON and DON) (0.330 vs. −0.398) (*P* ≤ 0.10). In other words, ingestion of diets supplemented with binder tended to be associated with higher gene expression of *OCLN*, *CLDN-1*, *CLDN-2*, *TNF-α* and *IAP* as compared to diet without binder. This finding is consistent with the gene expression result of *CLDN-1* in Table 4. It supports the finding that binder may also co-up-regulate the expression of other TJPs (*OCLN*, *CLDN-2*) and *IAP*. The second principal component indicates that the high expression of *TLR-4* was associated with high expression of pro-inflammatory cytokines *TNF-α* and *IL-1β*. The third principal component denotes that high expression of *CLDN-1*, *CLDN-2 *and *CLDN-5* was related to higher weight at d14. However, principal components 2 and 3 were not discriminatory for treatments.

**Table 5:** Loadings and principal component scores for 5 principal components obtained by principal component analysis ^a^ (PCA) of 11 variables from piglets fed diets with or without mycotoxin contamination, and with or without binder at d14 post-weaning

Treatment^b^	Principal component				
	1	2	3	4	5
	22.6%	22.2%	16.0%	15.6%	10.1%
CON	−0.225	0.119	0.119	0.719	0.517
CON + BIN	−0.019	0.006	0.390	−0.327	−0.455
DON	−0.571	0.630	−0.366	−0.477	0.119
DON + BIN	0.679	−0.630	−0.120	0.071	−0.151
*P*-value					
Mycotoxin	0.68	0.88	0.29	0.36	0.92
Binder	0.10	0.12	0.58	0.56	0.18
Mycot. × Binder	0.23	0.19	0.98	0.08	0.44
BW d14^c^			0.889		
FD4 permeability					0.974
OCLN	0.813				
CLDN-1	0.767		0.401		
CLDN-2	0.343		0.335	0.754	
CLDN-5		0.546	0.713		
TLR-4		0.891			
TNF-α	0.498	0.666			−0.300
IL-1β		0.866			
IL-8				0.910	
IAP	0.856				

^a^Rotation method: varimax with Kaiser normalisation; only correlations with |r| > 0.3 are indicated

^b^ *CON* negative control diet (uncontaminated basal diet), *CON + BIN* negative control diet with 1 kg/t mycotoxin binder, *DON* negative control diet with DON, *DON + BIN* negative control diet with DON and 1 kg/t mycotoxin binder. Principal components scores of subjects were analysed by General Linear model with fixed factor mycotoxin addition, binder addition and the interaction

^c^ *BW* body weight, *CLDN-1* Claudin 1, *CLDN-2* Claudin 2, *CLDN-5* Claudin 5, *FD4* FITC-dextran 4, *IAP* Intestinal alkaline phosphatase, *IL-1β* Interleukin 1 beta, *IL-8* Interleukin 8, *OCLN* Occludin, *TLR-4* Toll like receptor 4, *TNF-α* Tumor necrosis factor alpha

## Discussion

In the current experiment, mycotoxin contamination of piglet diets exhibited no effect on growth and gut health parameters. In contrast, the addition of a mycotoxin binder showed beneficial effects, in particular when diets were contaminated with 3 mg/kg of a mixture of DON and acetylated metabolites. Growth and feed intake were enhanced, in line with improvements of some selected gut health parameters.

### Lack of effect of DON addition to feed on performance and gut health

In addition to lack of effect on growth performance, our results did not show an effect of DON on gut health in the distal small intestine, regarding intestinal permeability and mRNA expression of TJPs and pro-inflammatory cytokines, as well as IAP, after 14 d of feeding 3 mg/kg total DON. The lack of effect in the distal small intestine might be associated with the toxicokinetic properties of DON. In vivo and in vitro studies demonstrated that DON and its acetylated forms are rapidly absorbed from the upper GIT, involving stomach until proximal jejunum. After chronic exposure to DON in pigs, a fast and almost complete absorption (> 90%) occurs, with DON appearing within 15 min in the blood and reaching maximal concentrations 1.65 h after oral exposure [22]. Danicke et al. revealed that 88.5% of the DON dose was detected in the stomach whereas only 1.5% in the small intestine [23]. Also, the acetylated derivatives of DON are rapidly hydrolysed to DON in vivo and then absorbed. Thus, the distal small intestine might be less susceptible to DON as the majority of DON is already absorbed in the proximal parts of the GIT [24]. All animal species can exhibit toxic effects when exposed to DON [3], with pigs being the most susceptible species [4]. However, the severity depends on various factors, including type and dose of DON, the route and duration of application, as well as the animal status [25]. After application of DON on the apical side or on the basolateral side of IPEC-J2 cells, Diesing et al. found that the apical epithelium seems to be more resistant to DON application while the same concentration of DON from basolateral side severely impairs barrier integrity [9]. In our case, it can be assumed that most DON was absorbed in the upper GIT, reaching the more susceptible basolateral side whereby only a small part of DON was left in the less susceptible apical side. That’s probably the reason why little effect of DON administration on gut health was seen in samples from the distal small intestine. Also, it should be taken into account that generally cytokine induction upon DON exposure occurs within hours of exposure [26], and thus differences after chronic exposure as in our study might not always be present.

So far, data about the effects of DON are incomplete, especially due to the lack of in vivo data. At first, feed naturally contaminated with DON was used in studies in vivo and in vitro. However, interpretation of data from naturally contaminated diets is complicated as co-occurrence with other mycotoxins is commonly found in cereals. Mycotoxicoses may be caused by multiple toxins, making it difficult to unravel the separate effects of the target mycotoxin DON. At present, it is challenging to study the effect of DON and its masked mycotoxins ADONs. Purified mycotoxins are generally used in in vitro experiments. In addition, it is difficult to correlate in vitro exposure with in vivo dosage as the amount of mycotoxin that can be absorbed in vivo does not necessarily correspond to the amount absorbed by cells in culture. Taken together, it’s difficult to conclude what dose DON will exhibit toxic properties, as the dose, the type of mycotoxin, the route and duration of exposure can all influence the mode of action.

### Binder addition to the feed improves performance and some parameters of gut health

The mycotoxin binder used in this study was a combination of acid-activated bentonite, clinoptilolite, yeast cell walls and organic acids and salt. Acid-activated bentonite increases the adsorption capacity by using a specific acid activation process to increase the surface area and to enlarge pores [27, 28]. Clinoptilolite, with a honeycomb like structure, serves to bind a broad range of mycotoxins [29]. Yeast cell walls, which contain *α*-*D*-mannans and *β*-*D*-glucans, have an active role in reducing mycotoxins in animal feed [30, 31]. Yeast cell walls used in the binder are extracted and harvested in the early stage of the fermentation, during which the network of covalent bonds is less dense, which offers more flexibility and a maximal accessibility of the mycotoxin binding sites [32–34].

In the current study, the ingestion of diets supplemented with binder reduced the expression of *TLR-4* compared to diets with no binder. TLR-4 plays an important role in recognizing Gram-negative bacteria and activation of the innate immune system. Activation of TLR-4 leads to the release of its downstream inflammatory modulators, including TNF-α and IL-1 [35]. This mechanism is well known and supported in the present study by the positive association between *TLR-4*, *TNF-α * and *IL-1β* mRNA expression that was evident in principal component 2 from the PCA. TLR-4 is most well-known for recognizing lipopolysaccharides (LPS), a structural component of the outer membrane of Gram-negative bacteria. LPS induce strong inflammatory responses in vivo, and are released when the cell is lysed or during bacterial cell division. Supplementation of toxin binders has shown reduced expression of pro-inflammatory cytokines such as IL-1β and IL-6 and other immune responses in LPS-induced pigs [36]. In other words, the mycotoxin binder might not only bind to mycotoxins, but might also bind other toxins such as bacterial endotoxins. This was not specifically investigated in the present study but might have occurred.

At the same time, there was a tendency that the supplementation of binder up-regulated the expression of *CLDN-1*. Claudins function as major components of the tight junction strands that regulate the permeability of epithelia. CLDN-1, a member of the claudin family, is an integral membrane protein. Enhanced *CLDN-1* expression can decrease paracellular permeability and tighten the tight junctions. As the mycotoxin binder in the current study may have adsorbed a range of toxins, it could be hypothesized that it adsorbed other xenobiotics which might impair the barrier function by deregulation of TJ assembly.

From the result of PCA, we know that groups with the supplementation of binder tended to have higher scores for principal component 1, which is positively associated with gene expression of *OCLN*, *CLDN-1*, *CLDN-2* and *IAP*, as compared to diets without binder. This finding is consistent with the gene expression result of *CLDN-1*. The PCA suggests that binder may also up-regulate the expression of other TJPs (OCLN, CLDN-2) and IAP. OCLD, together with the claudin group of proteins, is an important component of the tight junctions. Studies have shown that rather than being important in assembly and maintenance of tight junctions, OCLN is important in stability and barrier function of tight junctions. As the *OCLN* gene is essential and plays a fundamental role in modulating the epithelial tight junctions, OCLN is a respective marker of epithelial barrier and its presence or absence could reflect the permeability of intestinal epithelium [37]. Taken the correlation into consideration, the binder may stimulate the expression of other TJPs as OCLN is highly correlated to ZO-1, ZO-2 and CLDN-7. The family of ZO is a part of the cytoplasmic plaque of the TJPs. The importance of maintenance of gut barrier integrity is further illustrated by the grouping of weight at d14 and the mRNA levels of claudins within principal component 3.

IAP is a brush border enzyme which is a component of the gut mucosal defence system. IAP is involved in regulating secretion of bicarbonate in the duodenum. Failure to neutralize acid environment can lead to acidified chyme injuring epithelial cells, finally increasing inflammation and intestinal permeability. IAP is also known to detoxify LPS and prevent bacterial translocation in the gut [38]. As discussed, LPS will induce strong inflammatory responses in vivo. In other words, IAP can inhibit the inflammatory responses by detoxification of LPS. So, IAP is an important indicator to gut health.

Taken together, the addition of mycotoxin binder could improve the gut health by decreasing the expression of TLR-4 as well as increasing the expression of TJPs and IAP.

## Conclusions

The addition of a mycotoxin binder showed beneficial effects for weaned piglets, especially when diets were contaminated with 3 mg/kg of a mixture of DON in the pre-starter period. Growth and feed intake were enhanced. In line with this, reduced toll-like receptor-4 and increase of tight junction protein gene expression might shed light on the mode of action of the binder. However, the current study does not allow to assess whether the effects of the binder are mediated by alterations in the toxicokinetics of the mycotoxin.

## Abbreviations

15A–DON: 15-acetyl- deoxynivalenol
3A–DON: 3-acetyl- deoxynivalenol
AC: Activated carbon
ADFI: Average daily feed intake
ADG: Average daily gain
ADONs: Acetylated-deoxynivalenols
AFB1: Aflatoxin B1
CLDN-1: Claudin 1
CLDN-2: Claudin 2
CLDN-5: Claudin 5
CLDN-7: Claudin 7
DON: Deoxynivalenol
F:G: Feed to Gain Ratio
FB1: Fumonisin B1
FD4: FITC-dextran 4
GIT: Gastrointestinal tract
HCK: Hematopoietic cell kinase
HPRT-1: Hypoxanthine Phosphoribosyltransferase 1
HSCAS: Hydrated sodium calcium aluminosilicate
IAP: Intestinal alkaline phosphatase
IFN-γ: Interferon gamma
Ig: Immunoglobulins
IL-1β: Interleukin 1 beta
IL-8: Interleukin 8
LPS: Lipopolysaccharide
MAPK: Mitogen-activated protein kinase
NIV: Nivalenon
OCLN: Occludin
OTA: Ochratoxin A
PCA: Principal component analysis
PPIA: Peptidylprolyl isomerase A
SAPK/JNK: Stress-activated protein kinases/cJun N-terminal kinases
TBP: TATA-binding protein
TJP: Tight junction protein
TLR-4: Toll like receptor 4
TNF-α: Tumor necrosis factor alpha
ZEA: Zearalenone
ZO-1: Zona occludens 1
ZO-2: Zona occludens 2
